# “UKE CAN DO IT”: A REFLECTION ON A UKULELE CLASS INITIATIVE FOR OLDER ADULTS LIVING WITH DEMENTIA AND CARE PARTNERS

**DOI:** 10.1093/geroni/igae098.3067

**Published:** 2024-12-31

**Authors:** Joey Wong, Jason Fu, Mario Gregorio, Lillian Hung

**Affiliations:** University of British Columbia, Vancouver, British Columbia, Canada; University of British Columbia, Vancouver, British Columbia, Canada; University of British Columbia, Vancouver, British Columbia, Canada; University of British Columbia, Vancouver, British Columbia, Canada

## Abstract

Individuals with different stages of neurocognitive disorder or dementia respond to music even at later stages of the diagnosis. Music can offer collective experiences for people from diverse cultural backgrounds who have dementia. However, limited studies explore the journey of learning musical instruments by people living with dementia. Our team, co-led by a person living with dementia, initiated a 12-week basic ukulele program to explore the potentials of music and learning to foster social connections, alleviate loneliness and bring joy to older adults living with dementia and their care partners in Vancouver, British Columbia. Eighteen older adults from diverse backgrounds (15 individuals living with different stages of dementia and three care partners) participated in the classes facilitated by a ukulele instructor. Our team critically reflected on the ukulele program based on the in-class observations and summaries from the instructor through multiple in-person and Zoom meetings. The reflection resulted in five themes: 1) Social connections through music, 2) Respite for care partners, 3) Peer support and empowerment to contribute to collective learning experiences, 4) An emphasis on enjoyment over skills, and 5) Inclusiveness in creating programs for people living with dementia. This innovative ukulele program shows the positive impact of music and collective learning. The team reflections highlight the potential of offering musical instrument programs for older adults living with dementia and their care partners to alleviate loneliness and foster social connections. Future research can investigate the long-term impact of group musical learning on individuals living with dementia and care partners.

